# Fludarabine Downregulates Indoleamine 2,3-Dioxygenase in Tumors via a Proteasome-Mediated Degradation Mechanism

**DOI:** 10.1371/journal.pone.0099211

**Published:** 2014-06-09

**Authors:** Laïla-Aïcha Hanafi, Dominique Gauchat, Jessica Godin-Ethier, David Possamaï, Jean-Baptiste Duvignaud, Denis Leclerc, Nathalie Grandvaux, Réjean Lapointe

**Affiliations:** 1 Research Centre, Centre hospitalier de l′Université de Montréal (CRCHUM), Université de Montréal and Institut du Cancer de Montréal, Montréal, Québec, Canada; 2 Centre de recherche en infectiologie, Centre hospitalier universitaire de Québec and Department of Microbiology, Infectiology and Immunology, Université Laval, Québec, Québec, Canada; 3 PROTEO, Université Laval, Québec, Québec, Canada; 4 Research Centre, Centre hospitalier de l′Université de Montréal (CRCHUM) and Departement of Biochemistry, Université de Montréal, Montréal, Québec, Canada; University Hospital of Heidelberg, Germany

## Abstract

Indoleamine 2,3-dioxygenase (IDO) is found in multiple malignancies and exerts immunosuppressive effects that are central in protecting tumors from host T lymphocyte rejection. IDO is an enzyme involved in the catabolism of tryptophan resulting in inhibition of T lymphocyte function. While inhibition of IDO enzymatic activity results in tumor rejection, it is still unknown how we can directly target IDO expression within tumors using drugs. We have chosen to interfere with IDO expression by targeting the key-signaling event signal transducer and activator of transcription 1 (STAT1). We evaluated the efficacy of fludarabine, previously described to inhibit STAT1 phosphorylation. Interestingly, fludarabine was efficient in suppressing protein expression and consequently IDO activity in two different cell lines derived from breast cancer and melanoma when IDO was activated with interferon-gamma (IFN-γ) or supernatants prepared from activated T lymphocytes. However, fludarabine had no inhibitory effect on STAT1 phosphorylation. Other IFN-γ-responsive genes were only marginally inhibited by fludarabine. The level of IDO transcript was unaffected by this inhibitor, suggesting the involvement of post-transcriptional control. Strikingly, we have found that the inhibition of proteasome partially protected IDO from fludarabine-induced degradation, indicating that fludarabine induces IDO degradation through a proteasome-dependent pathway. Currently used in the clinic to treat some malignancies, fludarabine has the potential for use in the treatment of human tumors through induction of IDO degradation and consequently, for the promotion of T cell-mediated anti-tumor response.

## Introduction

Indoleamine 2,3-dioxygenase (IDO) is an enzyme involved in the catabolism of tryptophan affecting several immunoregulatory functions such as fetal protection [1], allograft tolerance and cancer progression [2]. IDO's immunosuppressive activity is due to decreased tryptophan availability and the generation of tryptophan metabolites, culminating in multi-pronged negative effects on T lymphocytes in proximity to IDO-expressing cells, such as inhibition of proliferation, effector functions and cell survival. IDO, which was developed as a key negative controller of anti-tumor T lymphocytes [3], is upregulated in response to activated T lymphocytes [4], and promotes the development of FoxP3^+^ regulatory T lymphocytes [5]. Consequently, inhibitors of IDO enzymatic activity have great therapeutic potential and some are currently being evaluated in clinical trials. The classic IDO inhibitor is 1-methyltryptophan (1-MT) [6]. However, it has been shown that the 1-d-MT isomer upregulates IDO1 in human cancer cells *in vitro* [7], and this upregulation can circumvent the enzymatic inhibitory effect of 1-MT. In addition, cancer cells may evolve to become resistant to this competitive inhibitor. Therefore, a more effective inhibitor is currently being tested in the clinic [8]. These approaches all target IDO activity directly, and there are only a few investigations aimed to target IDO expression pathways and stability.

IDO is usually expressed in antigen presenting cells such as dendritic cells and serves as a counter-regulatory mechanism to modulate immune responses [9]. Interferon-gamma (IFN-γ) has been identified as one of the main IDO inducers in multiple cell types [10]. We thus speculated that the IFN-γ signaling pathway leading to IDO expression could be targeted for altering IDO expression in the cancer microenvironment. Accordingly, different natural compounds can modulate IDO expression. For example, curcumin [11], green tea [12], resveratrol [13] and rosemary [14] can downregulate IDO by inhibiting the JAK-STAT kinase pathway. This pathway is thus an important target for modulation of IDO expression. In this report, we study fludarabine, a compound used for the treatment of some hematological malignancies such as chronic lymphocytic leukemia (CLL) [15]. Fludarabine has been shown to downregulate signal transducer and activator of transcription 1 (STAT1) activation [16]. In our study, we confirm fludarabine's effect on IDO protein levels and its activity in tumor cell lines. Interestingly, although fludarabine reportedly inhibits STAT1 phosphorylation in normal and cancer cells [17], [18], the signaling cascade leading to IDO expression remained unaltered in our system. We further established that fludarabine-mediated IDO downregulation occurs through a proteasome-dependent degradation pathway.

## Materials and Methods

### Ethics statement

This entire study including methods, obtaining of patient cell lines, blood and the written informed consent procedure was approved by the Ethics Committee of the Centre hospitalier de l′Université de Montréal (CHUM). Written informed consent was obtained from each healthy donor and patient prior to the collection of tumor specimens and blood samples. The patients and healthy donors have consent for their data to be use in research purposes. The consents and all other data were kept in confidentiality anonymously numbered.

### Normal donors, patients and cell lines

624.38mel cells were obtained from the NIH Surgery branch. 624.38mel is a clone selected from melanoma cell line 624 for its high expression of HLA-A2 molecules on its cell surface [19]. The written consent from the patient was obtained at the time of the establishment of the 624 cell line at the NIH Surgery branch. MDA-231 cell line was obtained from the ATCC. Both cell lines were cultured in RPMI 1640 supplemented with 10% fetal bovine serum, 2 mM l-glutamine, 100 U/ml penicillin, 100 µg/ml streptomycin, and 10 µg/ml gentamicin (all from Wisent). Heparinized blood, obtained from healthy donors by leukapheresis, was centrifuged on lymphocyte separation medium (Wisent) to isolate peripheral blood mononuclear cells (PBMC). Healthy donors were recruited by the Division of Hematology and Immunodeficiency Service of Royal Victoria Hospital (Dr Jean-Pierre Routy). Clinical samples were obtained from the *Banque de tissus et de données* of the *Réseau de recherche sur le cancer* of the *FRQ-S*, affiliated with the Canadian Tumour Repository Network (CTRNet). Freshly resected breast tumor samples were briefly stored in Iscove's modified Dulbecco's medium (Life technologies) prior to culture of tumor-infiltrating lymphocytes (TIL) as previously described [4].

### Reagents

l-tryptophan, 1-methyl d,l-tryptophan (1-MT) and kynurenine (all from Sigma-Aldrich) were prepared in distilled water. IFN-γ and interleukin (IL)-13 (Peprotech) were resuspended in Iscove's Modified Dulbecco's medium. Bortezomib (Selleck Chemicals), cycloheximide (Calbiochem) and fludarabine (TOCRIS Biosciences) were prepared in dimethylsulfoxide (DMSO). 6-thioguanine, azathioprine and 5-fluorouracil (all from Sigma) were all prepared at 50 mg/ml in NaOH 1M, NH_4_OH 1M and DMSO, respectively.

### Induction of IDO in cancer cell lines

Cancer cell lines were activated with 50 U/ml of IFN-γ, anti-CD3- or IgG2a-activated TIL or PBMC supernatants, as described previously [4]. Cells were harvested 30–60 min after activation for phospho-STAT1 and -STAT6 quantification, and after 24 h for IDO reverse transcriptase-polymerase chain reaction (RT-PCR), or IDO, STAT1, STAT6 and β-actin immunoblotting.

### RNA interference

STAT1 was silenced in MDA-231 cells by using small interfering RNA (siRNA) at 5 µM (STAT1-RNAi sense 5′-(P)CUACGAACAUGACCCUAUCUU-3′, anti-sense 5′-(P)GAUAGGGU CAUGUUCGUAGUU-3′, Dharmacon, Thermofisher Scientific) transfected with Dharmafect2 reagent according to the manufacturer' instructions. siGenome non-targeting RNApool2 (Dharmacon) served as controls at the same concentration. Proteins were extracted after 48 h and analyzed by immunoblotting.

### Assessment of IDO stability

MDA-231 cells were plated at 2×10^5^ cells/well in 12-well plate. Cells were then incubated for 24 h with 100 µM fludarabine or with DMSO as control (fludarabine pre-treatment). The cells were washed and incubated with indicated concentration of bortezomib or DMSO as control for 1 h. IDO was induced with 50 U/ml of IFN-γ. Approximately 24 h after activation, proteins were extracted and IDO revealed by immunoblot. To specifically assess IDO half-life, MDA-231 cells were plated at 2×10^5^ cells/well in 12-well plates and activated for 24 h with 50 U/ml IFN-γ. Cells were washed and treated with 100 µM of cycloheximide with or without 100 µM fludarabine. IDO protein stability was assessed between 0 and 24 h of incubation by immunoblot analysis.

### Proteasomal activity assay

MDA-231 were treated with various concentrations of fludarabine (10–200 µM) for 24 h with or without 50 U/ml of IFN-γ. Cells were harvested and plated at 2.5×10^4^ cells/well in white-bottom 96-well plates. Bortezomib was added at 50 nM as negative control for 1 h. Proteasome-Glo chymotrypsin-like cell-based assay reagent was added according to manufacturer instructions (Promega). Luminescence was quantified with the Synergy 4 microplate reader (BioTek).

### RT-PCR

RNA from cancer cell lines was extracted by RNeasy™ micro kit (Qiagen), according to the manufacturer' instructions. For quantitative RT-PCR analysis, cDNA was synthesized from mRNA with oligo-dT and random hexamers (both from Applied Biosystems), using the Omniscript reverse transcriptase kit (Qiagen). RT-PCR was performed as previously described [4].

### Immunoblotting

Proteins were prepared in the presence of HALT proteinase/phosphatase inhibitors (Thermofisher) from the above-mentioned pelleted cells, quantified, resolved on 10% SDS-PAGE, and transferred to polyvinylidene fluoride (PVDF) membranes (Bio-Rad). The membranes were incubated with anti-human IDO antibody (Ab) (Hycult Biotechnology) or anti-human β-actin-specific mouse monoclonal Ab (Chemicon) [4]. For total STAT1, and Y701 or S727 phosphorylated forms, proteins were resolved on 7.5% SDS-PAGE, incubated overnight at 4°C with rabbit polyclonal Ab (anti-STAT1, anti-Y701 and anti-S727 phospho-STAT1, all from Cell Signaling). IDO antibody specificity was confirmed using a plasmid encoding IDO which was transfected into MDA-231 cells using lipofectamine and plus reagent following manufacturer' instructions (Life technologies; Figure S1).

### IDO activity assay

IDO activity was evaluated as previously described [20]. Briefly, MDA-231 and 624.38mel were plated at 5×10^4^ cells/well in 48-well plates, treated for 24 h with 100 µM fludarabine or with DMSO as control, washed and stimulated with 50 U/ml IFN-γ. Approximately 24 h after activation, the cells were washed and re-suspended in Hank' balanced salt solution (HBSS; Wisent) containing 100 µM l-tryptophan and/or 500 µM 1-MT. Cells were then incubated for an additional 4 h, before harvesting of the supernatant and removal of cell debris by centrifugation. Tryptophan and kynurenine were quantified by high-performance liquid chromatography (HPLC) [20]. IDO activity was presented as micromolar concentration of kynurenine converted from tryptophan in samples.

### Flow cytometry

Non-specific binding sites were blocked with human gamma globulin (Jackson ImmunoResearch). Dead cells were eliminated from the analysis by staining with Live/Dead® Fixable Aqua Dead Cell Stain Kit (Life technologies). Cells were stained for 30 min at 4°C with the following fluorescent dye-conjugated monoclonal Ab – human leukocyte antigen (HLA)-A, -B, -C allophycocyanin (APC), and programmed cell death 1 ligand 1 (PD-L1), phycoerythrin-Cy7 (PE-Cy7) (both from BD Biosciences) – and washed in staining buffer (PBS containing 0.5% BSA and 0.1% NaN_3_). Flow cytometry data were acquired by LSR Fortessa cell analyzer with DIVA software (BD Biosciences). Mean fluorescence intensity (MFI) was calculated on cells stained positively with specific conjugated Ab.

## Results

### STAT1 is involved in IDO expression

**Figure 1:**
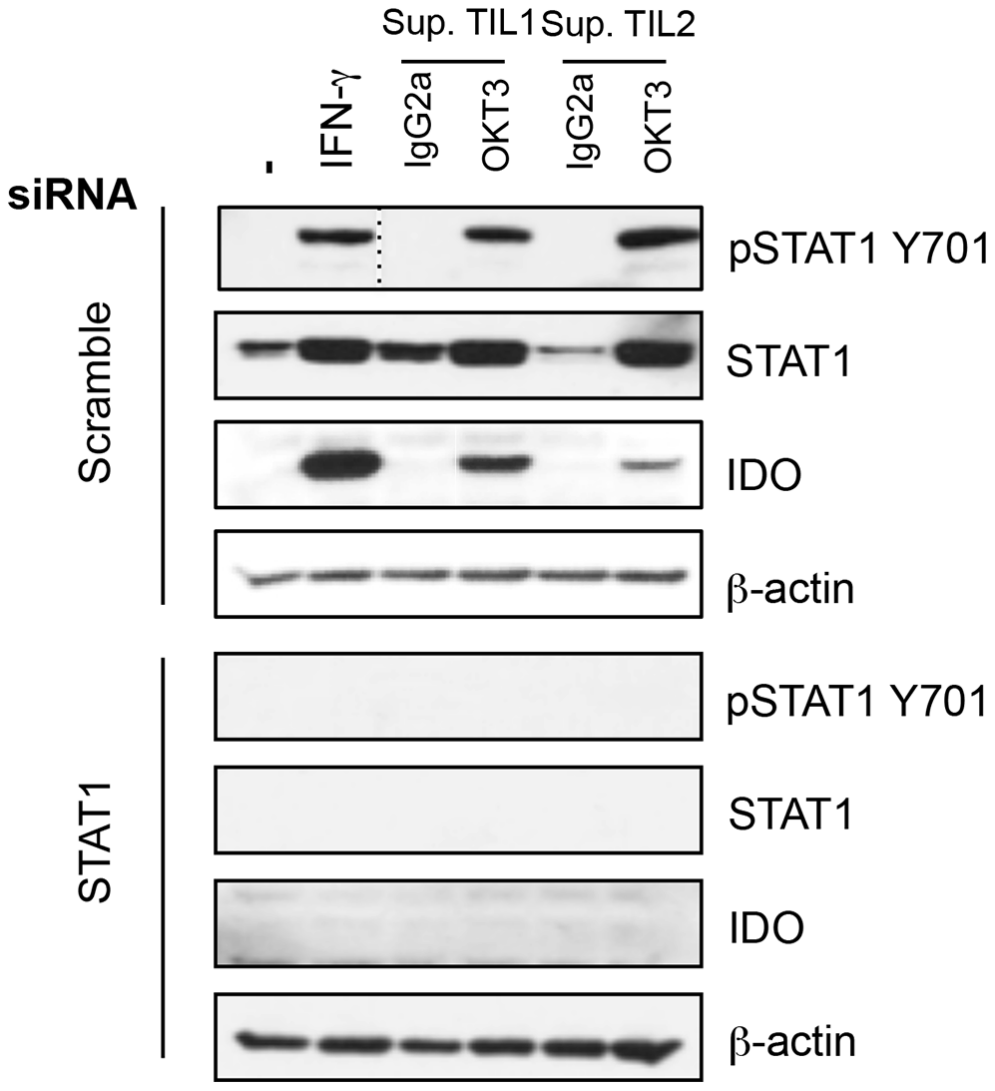
STAT1 is involved in IDO expression in response to T lymphocyte-derived factors. MDA-231 were transfected with siRNA against STAT1 or scrambled siRNA before activation with IFN-γ or supernatants of cultured TIL for 30 minutes (pSTAT1) or 24 h. Protein extracts were prepared for STAT1 (phosphorylated and total), IDO and β-actin immunoblot analysis. Results are representative of three independent experiments.

We evaluated whether the classical signaling pathways of IFN-γ (STAT1) and IL-13 (STAT6) were activated in tumor cells following culture in activated lymphocyte-conditioned medium. As shown in Figure S2, MDA-231 breast tumor cells exposed to IFN-γ or IL-13 induced phosphorylation of STAT1 and STAT6, respectively. However, only STAT1 was phosphorylated upon stimulation with activated T lymphocytes. To further mechanistically confirm the link between STAT1 and IDO expression, we silenced STAT1 in the MDA-231 cancer cell line by siRNA (Figure 1). In the absence of STAT1, IDO upregulation was abrogated after IFN-γ treatment or exposure to supernatants prepared from activated TIL. This was specific to STAT1 shutdown as IDO was normally upregulated under the same conditions with control (scrambled) siRNA. Therefore, our results confirm that inhibition of IDO expression can be obtained through abrogation of STAT1 engagement.

### Fludarabine decreases IDO expression through a STAT1-independent process

**Figure 2:**
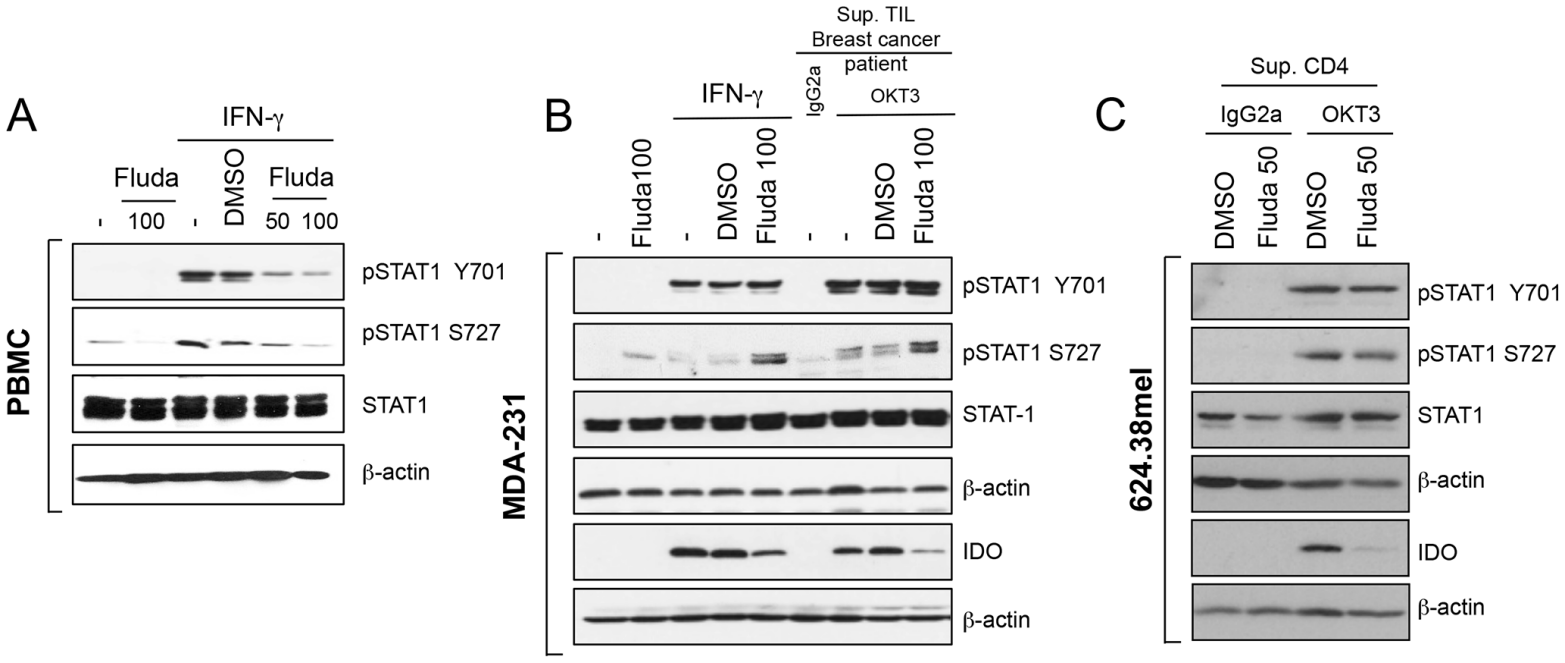
Fludarabine inhibits IDO protein independently of STAT1 phosphorylation on Y710 and S727. **A-** PBMC were pre-treated with the indicated concentrations of fludarabine or DMSO (vehicle) for 24 h. Cells were washed and activated for 30 min (pSTAT1) or 24 h (total STAT1 and β-actin) with 50 U/ml of IFN-γ. **B-** MDA-231 were pre-treated with 100 µM fludarabine before activation with 50 U/ml of IFN-γ, anti-CD3 (OKT3) or IgG2a-activated TIL supernatants (Sup.). **C-** 624.38mel were pre-treated with 50 µM fludarabine, and cultured with anti-CD3 (OKT3) or IgG2a-activated CD4^+^ T lymphocyte supernatants. **B-C** Cells were harvested after 30 min (pSTAT1, STAT1 and β-actin) or 24 h (IDO and β-actin). **A-C** Proteins were extracted for immunoblot analysis. Results are representative of three independent experiments.

Fludarabine was previously found to inhibit STAT1 phosphorylation in stimulated PBMC (Figure 2A) [16] and in smooth muscle cells [17], [21] as well as in renal cell carcinoma [18]. Interestingly, when we pre-treated the MDA-231 breast cancer cell line with a similar amount of fludarabine, IDO upregulation by IFN-γ was reduced (Figure 2B). Furthermore, fludarabine also inhibited IDO expression in a breast cancer cell line exposed to supernatants prepared with anti-CD3-activated TIL from two different breast cancer samples (Figure 2B) and in a melanoma cell line treated with supernatants prepared from anti-CD3-activated T lymphocytes (Figure 2C).

We evaluated whether IDO inhibition by fludarabine occurred at the stage of STAT1 phosphorylation. Unexpectedly, fludarabine treatment had no consistent effect on STAT1 protein expression level and phosphorylation (Figure 2). When we pre-treated MDA-231 (Figure 2B) and 624.38mel (Figure 2C) with fludarabine, we consistently observed the loss of IDO upregulation in response to IFN-© or activated T lymphocyte supernatants. However, fludarabine had no negative effect on STAT1 phosphorylation at either Y701 or S727 sites (Figure 2B-C). In fact, we observed an upregulation of S727 phosphorylation in MDA-231 by fludarabine treatment in presence or absence of IFN-© stimulation (Figure 2B).

### Fludarabine inhibits IDO activity independently of mRNA levels

**Figure 3:**
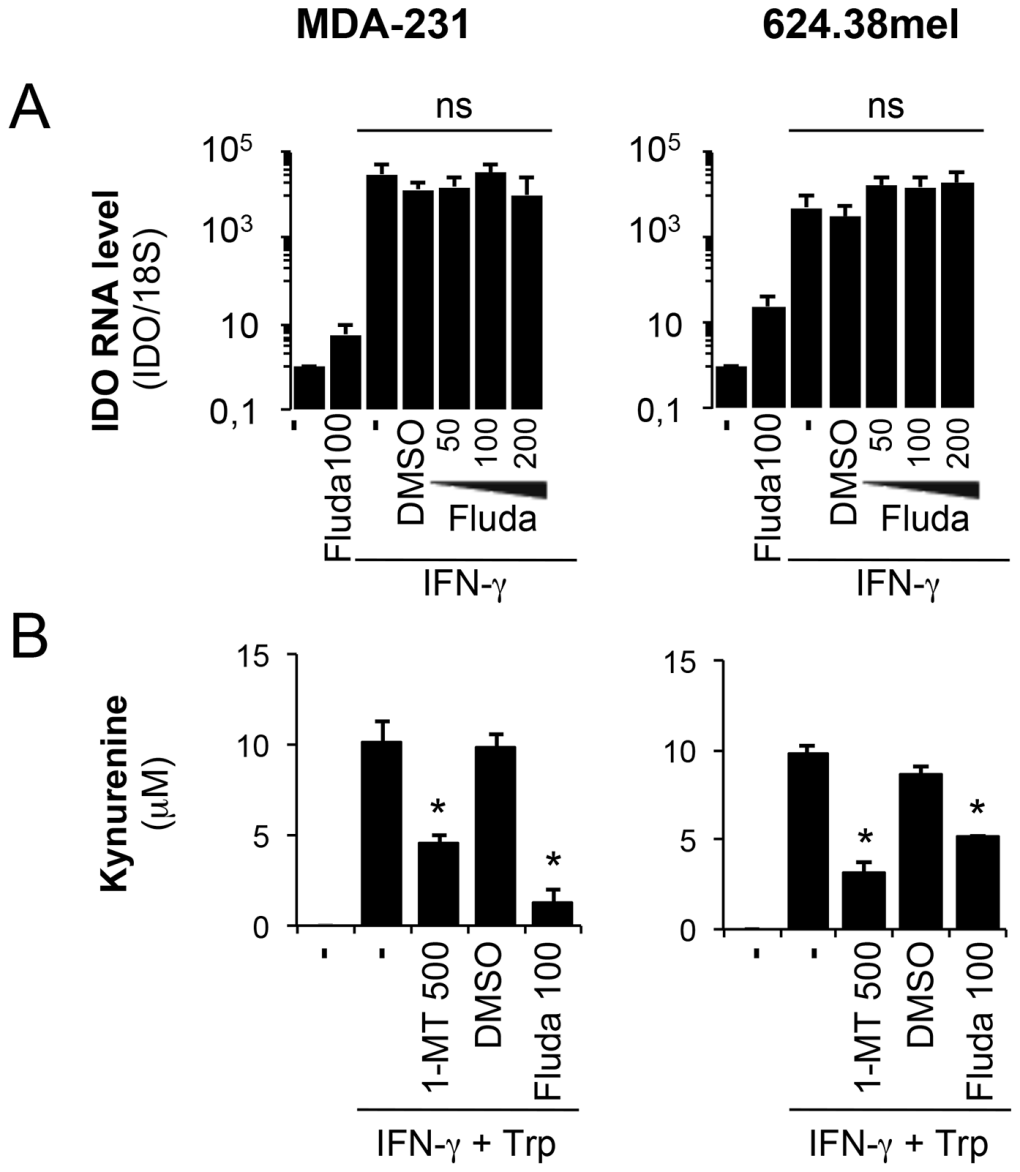
Fludarabine inhibits IDO activity independently of the mRNA level. **A-** MDA-231 and 624.38 pre-treated with 100 µM of fludarabine or DMSO prior to IFN-γ activation with 50 U/ml for 24 h. RNA was extracted from activated cells. cDNA was prepared and IDO expression was evaluated by quantitative real-time RT-PCR and normalized to 18S rRNA. Error bars represent standard deviation. Results are representative of three independent experiments **B-** MDA-231 and 624.38 were pre-treated with 100 µM of fludarabine or DMSO prior to IFN-γ activation with 50 U/ml for 24 h. Cells were resuspended in HBSS with tryptophan with or without 1-MT and incubated for 4 h. Kynurenine was quantified by HPLC. Errors bars represent standard deviation of triplicates of one experiment. * p<0.05 t-test compared to IFN-© without inhibitor. Results are representative of two independent experiments.

We then investigated whether fludarabine impacts IDO at the transcriptional level. As depicted in Figure 3A, IDO mRNA was clearly upregulated by IFN-© as expected, but fludarabine had no inhibitory effect, demonstrating that it does not affect IDO at the transcriptional level or during upstream IDO signaling events. This further suggests that fludarabine affects IDO at the protein level.

We next evaluated whether this decrease in IDO protein levels correlates with decreased IDO enzymatic activity. MDA-231 and 624.38mel were again pre-treated with fludarabine, and then exposed to IFN-©. IDO activity was assessed by evaluating the production of kynurenine, a metabolite of tryptophan degradation by IDO. As shown in Figure 3B, fludarabine had a strong inhibitory effect on kynurenine production. Our positive control, 1-MT, a classical inhibitor of IDO enzymatic activity [6], also has a negative effect on kynurenine production (Figure 3B).

In summary, fludarabine had a clear limiting effect on IDO upregulation and activity in response to stimulated T lymphocytes and IFN-©, but not one acting via the STAT1 signaling pathways (expression or phosphorylation) or at the level of IDO transcription.

### Expression levels of other IFN-γ-responsive genes are not affected by fludarabine

**Figure 4:**
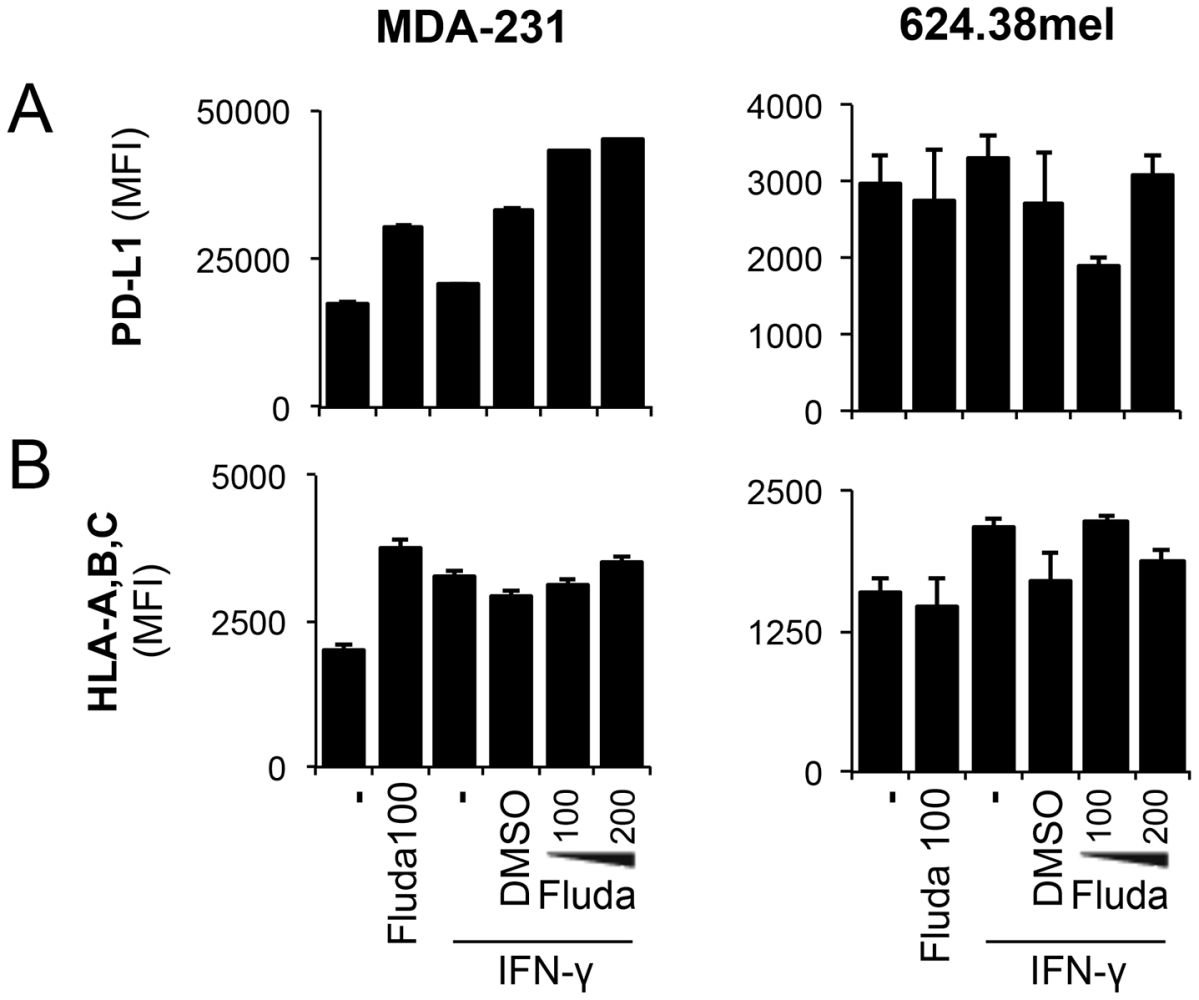
MHC I and PD-L1 expression levels remain unchanged following fludarabine treatment. MDA-231 and 624.38mel were pre-treated with the indicated concentrations of fludarabine or DMSO prior to IFN-γ activation with 50 U/ml for 24 h. Cells were harvested for flow cytometry analysis. MFI was assessed on viable populations for **A-** PD-L1 and **B-** HLA-ABC. Error bars represent standard deviation from one experiment. Results are representative of three independent experiments.

We next investigated whether fludarabine affects the expression of other IFN-©-responsive genes, such as the MHC I and PD-L1 genes [22]. Surprisingly, fludarabine had no negative effect on PD-L1 expression in MDA-231 and 624.38mel cells (Figure 4A). Furthermore, MHC I expression, as evaluated using a pan-specific antibody, was not negatively impacted by fludarabine in MDA-231 (Figure 4B), despite increases being observed in 624.38mel.

### The downregulation of IDO by fludarabine is higher compared to other nucleoside analogs

**Figure 5:**
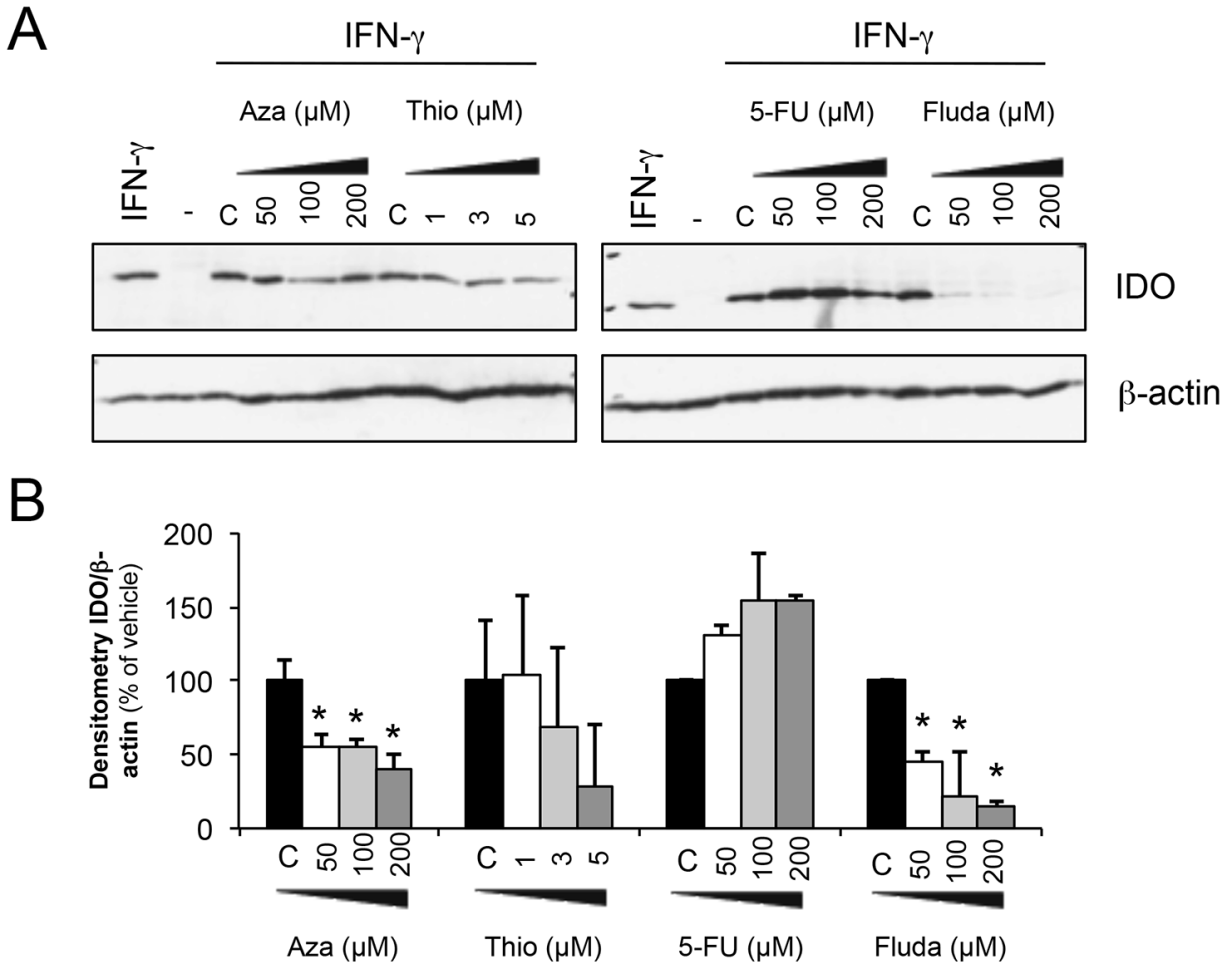
Downregulation of IDO by nucleoside analogs. **A-** MDA-231 were pre-treated with various nucleoside analogs at indicated concentrations or with vehicle of each inhibitor for 24 h. Cells were washed and 50 U/ml of IFN-γ were added. Proteins were extracted after 24 h for immunoblot analysis. **B**- Compilation of densitometry analysis of three different immunoblots as described in **A**. * significantly lower expression of IDO compared to vehicle (p<0.05 t-test).

We next evaluated whether other nucleoside analogs had similar effects on IDO. As presented in Figure 5, 6-thioguanine [23], azathioprine [24] and 5-fluorouracil [25] were tested at three different documented concentrations, though none of these showed an inhibitory effect on IDO protein levels that was comparable to that by fludarabine. However, a significant downregulation of IDO protein expression was noted with azathioprine (Figure 5B).

**Figure 6:**
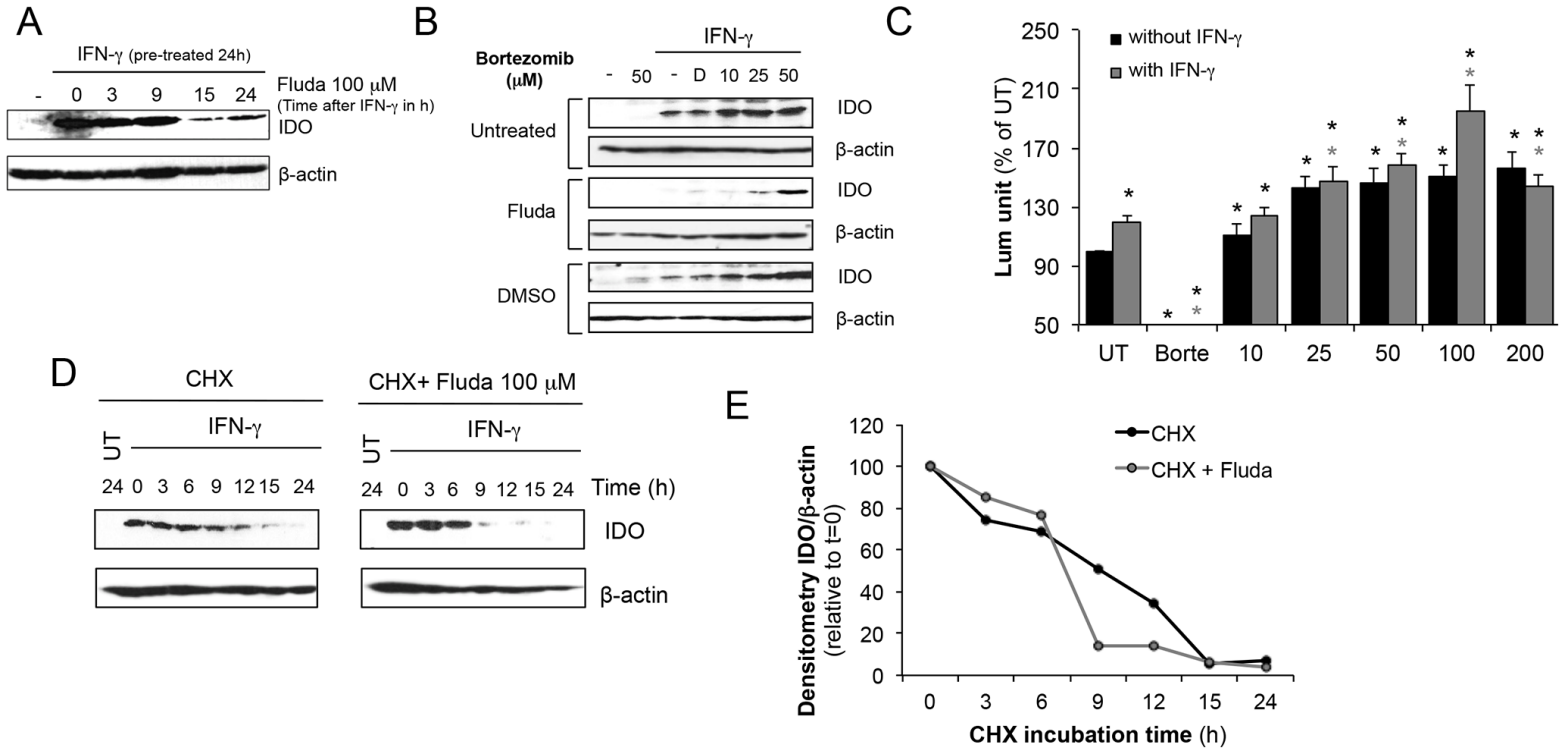
Fludarabine inhibits IDO via a proteasome-mediated degradation pathway. **A-** MDA-231 cells were first activated with IFN-γ (50 U/ml) to induce IDO expression for 24 h. Cells were then washed and treated with 100 µM of fludarabine for 3-24 h. Proteins were extracted for Immunoblot analysis. Immunoblots are representative of three independent experiments **B-** To evaluate the role of the proteasome, MDA-231 cells were pre-treated with 100 µM of fludarabine for 24 h. Cells were washed and treated with indicated concentrations of bortezomib before IFN-γ (50 U/ml) activation. Proteins were extracted after 24 h for immunoblot analysis. D: DMSO control (bortezomib vehicle). Bortezomib concentrations are expressed in nM. Immunoblots are representative of four independent experiments **C-** Cells were pretreated with indicated concentration of fludarabine with or without 50 U/ml of IFN-γ and proteasomal activity was assessed using Proteasome-Glo kit. Results are represented as % of activity of untreated cells. Results combine data from 6 independent experiments and 3 independent experiments with IFN-γ. Black * p<0.01 t-test compared to untreated; grey * p<0.01 t-test compared to untreated with IFN-γ. **D-E** 50 U/ml of IFN-γ were used to activate the cells to express IDO. 24 h later, cells were washed and incubated with 100 µM of cycloheximide with or without 100 µM fludarabine. IDO protein stability was assessed at indicated time by immunoblot. Results are representative of three independent experiments. **E**- Densitometry of IDO/actin by immunoblot in **D**.

### Fludarabine leads to IDO degradation through a proteasome-dependent pathway

We first determined if fludarabine inhibitory effect on IDO protein expression could be obtained after induction with IFN-γ. We upregulated IDO with IFN-γ for 24 hours and then treated with fludarabine. Similarly to our pre-treatment experiments, we observe a decrease in IDO starting at 15 hours of post-treatment with fludarabine (Figure 6A). To evaluate the effects of fludarabine on IDO stability we performed experiments by treating MDA-231 with a proteasome inhibitor (bortezomib [26], [27]). Cells were pre-treated with fludarabine or DMSO as a control. They were then stimulated with IFN-©in presence or absence of bortezomib, and IDO protein levels were analyzed by immunoblotting. As illustrated in Figure 6B, bortezomib restored IDO protein in a dose-dependent manner in fludarabine pre-treated cells. Also, bortezomib appeared to enhance IDO induction by IFN-© in the absence of fludarabine, indicating that a proteasome-dependent degradation process finely regulates IDO stability. This degradation is increased in the presence of fludarabine [28]. We investigated whether fludarabine directly impacted proteasome activity. For this, we performed a chemiluminescence-based assay of proteasome activity, and detected an upregulated proteasomal activity in MDA-231 cells pre-treated with various fludarabine concentrations with or without IFN-γ (Figure 6C). This upregulation was measured at all concentrations in absence of IFN-γ. In addition, we observed a fludarabine-dependent upregulation of proteasome activity with the IFN-γ treatment as compared to untreated cells, and further upregulation using fludarabine at 100 µM in presence of IFN-γ. Finally, we performed an IDO protein stability assay to assess the accelerated degradation rate in presence of fludarabine. In Figure 6D-E, we observed that IDO protein became undetectable by immunoblotting at 15 h when using cycloheximide to block translation. In presence of fludarabine, this threshold was reached after only 9 h. We therefore conclude that fludarabine reduces IDO stability by accelerating its degradation.

## Discussion

In this study, we aimed to inhibit IDO expression induced by IFN-γ by targeting STAT1 signaling in cancer cell lines. We first confirmed that IDO expression in our cancer cell line model was dependent on STAT1 signaling following exposure to IFN-γ or CD3-activated T lymphocyte supernatant. The use of fludarabine, a known inhibitor of STAT1 phosphorylation, strikingly reduced IDO protein levels. However, this inhibition was STAT1-independent. We also demonstrated that the inhibition was indeed at the protein level, because IDO mRNA was not affected by fludarabine. Finally, we established that IDO was sensitive to proteasomal degradation and that fludarabine enhanced such degradation leading to reduced protein levels.

First, we demonstrated the importance of STAT1 in IFN-γ signaling upstream of IDO expression in tumor cell lines. We have previously demonstrated that IDO expression in tumor cell lines is under the control of cytokines produced by activated T lymphocytes and that IL-13 has the capacity to repress IFN-γ-dependent induction of IDO [4]. It is thus clear that STAT1 is phosphorylated in tumors in response to IFN-© but also when exposed to activated T lymphocytes. We concluded that the STAT1 is the dominating pathway in cancer cell lines following their interaction with activated T lymphocytes.

Furthermore, although IDO expression depends on IFN-γ in multiple cell types, this is the first demonstration of STAT1 signaling involvement in a tumor cell line. Interestingly, STAT1 phosphorylation has been previously reported to be essential for IDO expression in CD19^+^ splenic dendritic cells in mice, but not following IFN-γ stimulation [29]. Still, we wanted to inhibit STAT1 phosphorylation and impair signaling leading to IDO expression with a chemical compound suitable for clinical application. To specifically target the STAT1 pathway, we chose fludarabine, a purine analog used as a chemotherapeutic agent in CLL [30] and hematopoietic tumors. It is also used as part of the cocktail in combination with cyclophosphamide as a non-myeloablative lymphodepletive regimen prior to the efficient treatment of melanoma patients by adoptive TIL transfer [31], [32]. Fludarabine was initially used in this study for its inhibitory effect on STAT1 phosphorylation in other cell types [16]–[18]. However, the results showed that IDO is downregulated in a STAT1-independent manner, which contradicts previous reports in a human lens epithelial cell line where fludarabine suppressed STAT1 phosphorylation [33]. Interestingly, another group has already reported that STAT1 expression was not affected by fludarabine in PBMC from CLL patients [34]. This confirmed that certain neoplastic cells respond differently to fludarabine treatment. In this study, we demonstrated a reproducible post-transcriptional inhibition of IDO with fludarabine in MDA-231 and 624.38mel cells, and also in the kidney cancer cell line, KTCL (Figure S3).

Finally, the mechanism of IDO inhibition by fludarabine may be due to an enhanced proteasomal activity. Control of IDO levels has previously been observed in the context of SOCS3 signaling leading to proteasomal degradation [28], [35]. Also, sodium butyrate has been described to inhibit STAT1 signaling and to upregulate IDO ubiquitination in human nasopharyngeal cancer cell lines [36]. While the main strategy currently envisioned to tackle IDO clinically is by inhibiting its enzymatic activity [37], our study demonstrates that IDO is sensitive to proteasomal degradation, which can be enhanced with fludarabine. Enhancing IDO degradation is an interesting alternative and a complementary approach to the enzymatic inhibition of IDO. Our study has established that inhibition of IDO is induced by IFN-γ stimulation. It will be interesting to define the impact of fludarabine on cancer cells constitutively expressing IDO, since the inhibition we report herein is a post-transcriptional one. Imatinib was used to repress constitutively expressed IDO in GIST [38], and fludarabine may be also useful in this context. Furthermore, as IDO also displays regulatory signaling capacity independent of its enzymatic activity [39], [40], targeting IDO at the protein level not only reduces its enzymatic activity, but also prevents the long-term immune-suppressive impact of IDO-driven TGF-β signaling.

In conclusion, considering that fludarabine impairs T cell dependent IDO upregulation, its use in pre-treatment of patients prior to immunotherapies involving a T cell response can potentially prevent IDO upregulation, thereby curtailing this immuno-suppressive mechanism. Our data suggest that fludarabine used in the context of non-myeloablative lymphodepletive regimens in multiple immune-therapeutic protocols [41], [42] may involve a blockage of IDO activity through proteasomal degradation. The combination of fludarabine with other IDO inhibitors, and with other immune-checkpoint modulators may dramatically decrease immune-suppression in immunotherapies. Such a complete IDO shutdown may be critical in controlling anti-tumor immunity, especially in considering the recent impact of immune-checkpoint modulators in clinical trials [43], [44].

## Supporting Information

Figure S1 IDO antibody specificity assessement MDA-231 were transfected with plasmids encoding GFP, or IDO or irrelevant control protein. As a control, MDA-231 were untransfected and untreated (UT) or stimulated with IFN-γ for 24h. Cells were harvested and proteins were prepared for IDO and β-actin immunoblot analysis.
(TIF)

Figure S2 STAT1 phosphorylation induction by PBMC supernatants MDA-231 were treated with IFN-γ, IL-13 or supernatants of cultured CD4^+^ or CD8^+^ T lymphocytes from healthy donors PBMCs for the indicated time (30 or 60 min). Cells were harvested and protein extracts were prepared for STAT1 (total and pY701), STAT6 (total and phosphorylated) and β-actin immunoblot analysis.
(TIF)

Figure S3 IDO inhibition by fludarabine is reproduced in kidney cancer cell line **A**- KTCL were pre-treated with 100 µM of fludarabine or DMSO prior to IFN-γ activation with 50 U/ml for 24 h. Cells were resuspended in HBSS with tryptophan with or without 1-MT and incubated for 4 h. Kynurenine was quantified by HPLC. Errors bars represent standard deviation of triplicates of an experiment. **B**- KTCL were pre-treated with the indicated concentrations of fludarabine or DMSO prior to IFN-γ activation with 50 U/ml for 24 h. Cells were harvested for flow cytometry analysis. MFI was assessed on viable populations for HLA-ABC. **C**- KTCL pre-treated with 100 µM of fludarabine or DMSO prior to IFN-γ activation with 50 U/ml for 24 h. RNA was extracted from activated cells. cDNA was prepared and IDO expression was evaluated by quantitative real-time RT-PCR and normalized to 18S rRNA. Error bars represent standard deviation. Representative of three independent experiments.
(TIF)
