# Supplementary figures and images for: Fludarabine Downregulates Indoleamine 2,3-Dioxygenase in Tumors via a Proteasome-Mediated Degradation Mechanism
Source: PLoS One. 2014 Jun 9;9(6):e99211. doi: 10.1371/journal.pone.0099211 (PMC4050125; doi:10.1371/journal.pone.0099211)

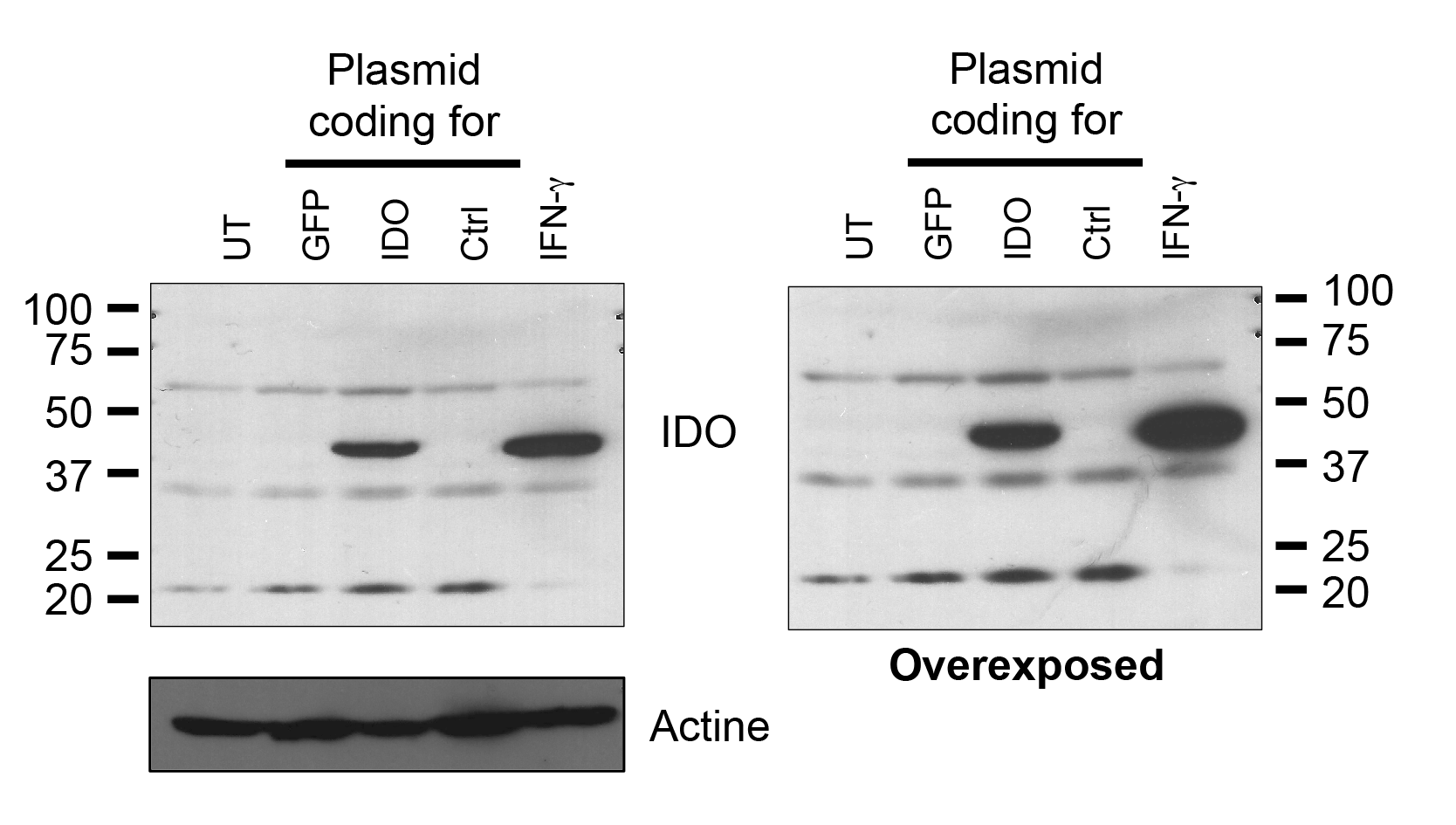

Supplement: Figure S1 — IDO antibody specificity assessement MDA-231 were transfected with plasmids encoding GFP, or IDO or irrelevant control protein. As a control, MDA-231 were untransfected and untreated (UT) or stimulated with IFN-γ for 24h. Cells were harvested and proteins were prepared for IDO and β-actin immunoblot analysis. (TIF) [file pone.0099211.s001.tif]

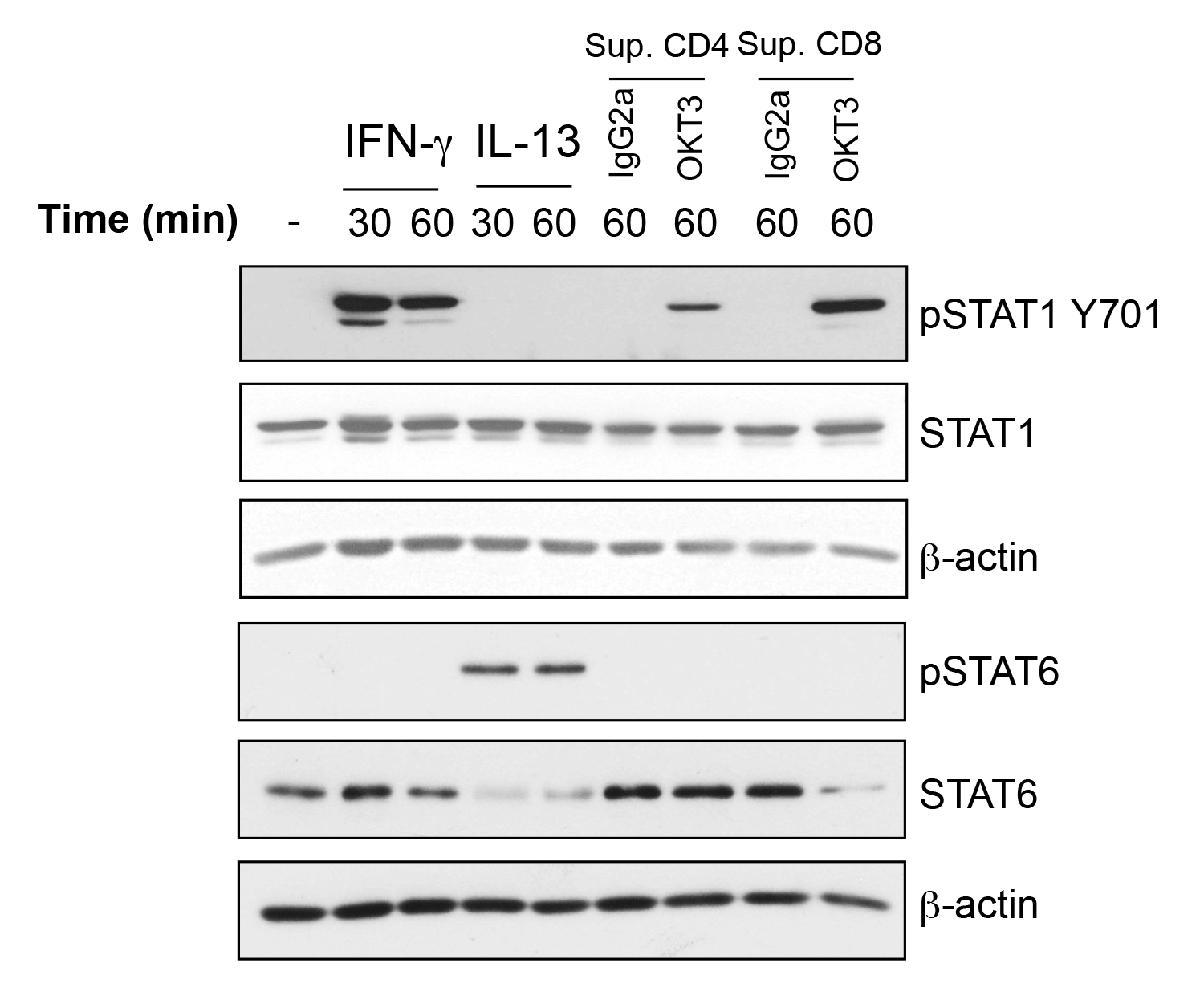

Supplement: Figure S2 — STAT1 phosphorylation induction by PBMC supernatants MDA-231 were treated with IFN-γ, IL-13 or supernatants of cultured CD4+ or CD8+ T lymphocytes from healthy donors PBMCs for the indicated time (30 or 60 min). Cells were harvested and protein extracts were prepared for STAT1 (total and pY701), STAT6 (total and phosphorylated) and β-actin immunoblot analysis. (TIF) [file pone.0099211.s002.tif]

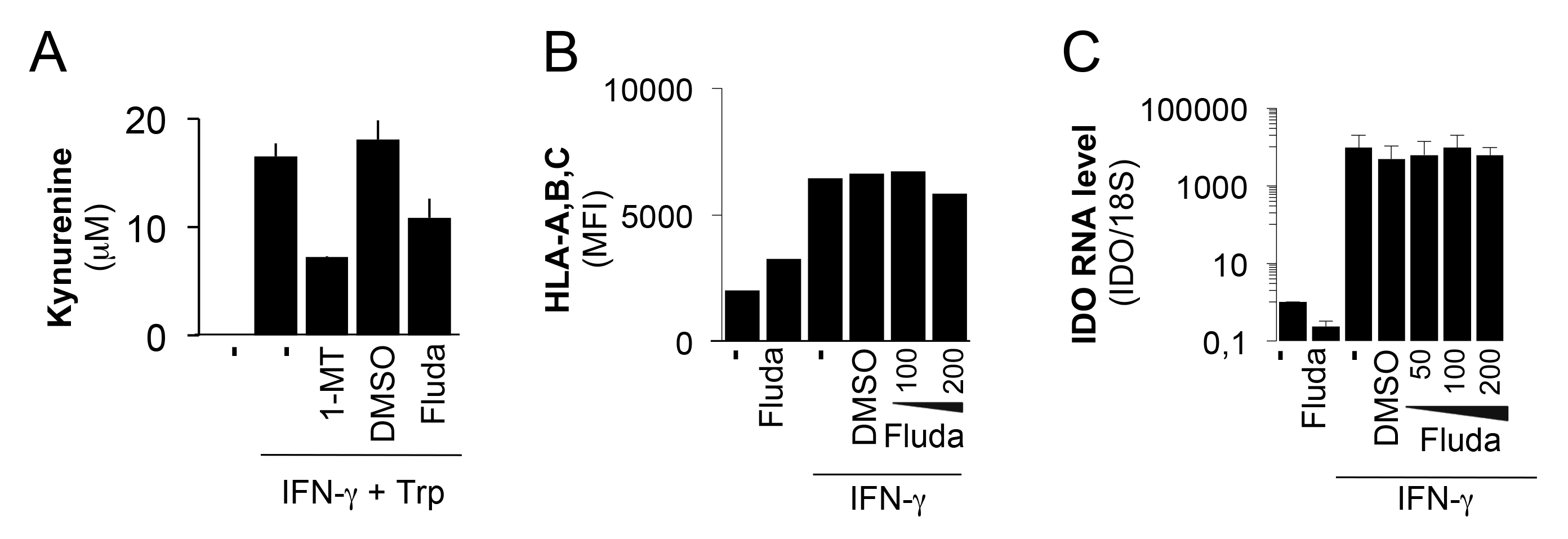

Supplement: Figure S3 — IDO inhibition by fludarabine is reproduced in kidney cancer cell line A- KTCL were pre-treated with 100 µM of fludarabine or DMSO prior to IFN-γ activation with 50 U/ml for 24 h. Cells were resuspended in HBSS with tryptophan with or without 1-MT and incubated for 4 h. Kynurenine was quantified by HPLC. Errors bars represent standard deviation of triplicates of an experiment. B- KTCL were pre-treated with the indicated concentrations of fludarabine or DMSO prior to IFN-γ activation with 50 U/ml for 24 h. Cells were harvested for flow cytometry analysis. MFI was assessed on viable populations for HLA-ABC. C- KTCL pre-treated with 100 µM of fludarabine or DMSO prior to IFN-γ activation with 50 U/ml for 24 h. RNA was extracted from activated cells. cDNA was prepared and IDO expression was evaluated by quantitative real-time RT-PCR and normalized to 18S rRNA. Error bars represent standard deviation. Representative of three independent experiments. (TIF) [file pone.0099211.s003.tif]
